# ASPRONET: A facilitated online education project for radiation therapists in the Asia-Pacific region

**DOI:** 10.1016/j.tipsro.2024.100283

**Published:** 2024-10-18

**Authors:** Craig Opie, Aidan Leong, Chetana Vartak, Iain Ward, Sandra Ndarukwa

**Affiliations:** a Northern Sydney Cancer Centre, Royal North Shore Hospital, Sydney, Australia; b School of Dentistry And Medical Sciences, Charles Sturt University, Wagga Wagga, Australia; c School of Primary and Allied Health Care, Monash University, Melbourne, Australia; d Bowen Icon Cancer Centre, Wellington, New Zealand; e Department of Radiation Therapy, University of Otago, Wellington, New Zealand; f Department of Radiation Oncology, Tata Memorial Hospital, Mumbai, India; g Canterbury Regional Cancer and Haematology Service, Christchurch Hospital, Canterbury District Health Board, New Zealand; h Department Of Medicine, University of Otago, Christchurch, New Zealand; i Applied Radiobiology and Radiotherapy Section, Division of Human Health, International Atomic Energy Agency, Vienna, Austria

**Keywords:** Radiation oncology, Asia-Pacific region, Radiation therapist, Videoconferencing, Clinical education

## Abstract

In 2019, the International Atomic Energy Agency approved a technical co-operation project, aimed at supporting clinical decision making and continuing professional education of radiation oncologists, medical physicists and radiation therapists (RTs) in Low-and-Middle Income Countries (LMICs) in the Asia Pacific region. From this, the Asia-Pacific Radiation Oncology Network (ASPRONET) was formed in 2020. An RT co-ordination group administered 16 online, one-hour seminars between December 2021 and November 2023 for an RT audience. Analysis of online registration and attendance data from each seminar was used to co-ordinate group review meetings, improve seminar proceedings, and promote attendance and engagement. 772 attendees from 20 different countries were recorded in total across the seminars. Gathered data and observations indicated the success of the seminars and supported their continuation.

## Introduction

Radiation therapy is an essential cancer treatment modality. Global demand has seen a fivefold increase in the number of radiation oncology centres since 1980.[1] A substantial portion of this growth has taken place in the Asia-Pacific region where cancer incidence is rising,[2] and which comprises 52.4 % of the world’s population.[3], [4] Safe and effective radiation therapy is contingent on the technical knowledge and coordinated clinical practice of three key professional groups – radiation oncologists (ROs) radiation oncology medical physicists (ROMPs) and radiation therapists (RTs). The importance of clinician training to impart and maintain technical knowledge in radiation therapy was highlighted in a theme mapping exercise of seven international documents relating to improving safety and quality in radiation therapy, in which it ranked highest.[5] The International Atomic Energy Agency (IAEA) has committed to this endeavour by administering a large range of educational materials and technical co-operation projects for radiation therapy.[6] RAS RCA 6096 (“ASia-Pacific Radiation Oncology NETwork; ASPRONET)” is an educationally oriented IAEA technical co-operation project, which commenced in 2020. The project aims to empower clinicians in Asia-Pacific region Low-and-Middle Income Countries (LMICs), to raise standards of care, improve survival and quality of life for cancer patients. Central to the project are concurrent meetings and education sessions for ROs, ROMPs and RTs working in LMICs in the Asia-Pacific region. This paper describes the development and administration of the ASPRONET RT program over its first two years, and reports on the learnings of the RT project leadership team from the seminar series.

## Methods

### Project formation

The development of ASPRONET followed an IAEA project: the AFrica Radiation Oncology NETwork (AFRONET), which sought to reduce observed barriers to generation and maintenance of technical expertise for clinicians in African LMICs. [7], [8], [9] Commencing in 2012, and drawing on the emerging successes of online clinical education approaches,[10]. It consisted of monthly virtual tumour board (VTB) meetings among African LMIC centres, using videoconferencing facilities. Of the 30 nations which the World Bank classifies as LMICs in South Asia, East Asia and the Pacific, 21 are IAEA member states. [11], [12] In 2019, following the successes of the AFRONET project [13] a core group of clinicians from the region submitted a proposal to the IAEA to create a similar VTB project for Asia-Pacific region member states. 18 participating nations nominated national project co-ordinators (NPCs) in mid-2020 to plan a VTB series, which commenced in November 2020. In July 2021, invitations for ROMP and RT representatives were issued. An RT leadership team from New Zealand and Australia began planning the format and operational details of the online RT education seminar series in September 2021, which went on to commence in December 2021.

### Seminar format

A total of 16 seminars were conducted in two phases of the series. For the first phase the RT leadership team devised a schedule of nine monthly seminars, which each had a distinct anatomical site theme, shown in Table 1. They created a Microsoft PowerPoint® slide template, a set of common topic categories, an online registration form and a promotional strategy. The RT leadership team were joined by a third member from India in March 2022. They met regularly to review each seminar, and to plan for the next in the series. Seminars were conducted live, in English, on Fridays at 05:00 to 06:00 UTC, with the RT leadership team chairing the seminars and guiding proceedings. Attendance data was gathered from Microsoft Teams®. The format was intended to facilitate learning through knowledge sharing among attendees, avoiding didactic approaches, and was targeted to accommodate the needs of LMIC RTs.

**Table 1:** ASPRONET RT Seminars − Major Themes, Dates, Registrations And Attendances.

**Phase 1 – Anatomical Themes**			
**Theme**	**Date**	**Registrations**	**Attendances**
Brain	10/12/2021	25	25
Head And Neck	14/01/2022	4	4
Breast	11/02/2022	29	17
Lung	11/3/2022	48	20
Abdomen	08/04/2022	83	36
Pelvis − Gynaecological	13/05/2022	58	26
Pelvis − Genito-urinary	10/06/2022	69	37
Pelvis − Colorectal	08/07/2022	49	13
Extremities	12/08/2022	39	12
**Phase 2 – Complex Technical Practice Themes**			
IGRT Decision Making	26/11/2022	149	51
DIBH	14/1/2023	163	75
Brachytherapy	11/3/2023	212	67
Stereotactic RT	13/05/2023	416	189
Clinical Education	08/07/2022	149	27
Paediatric RT	09/09/2023	214	87
RT Role Evolution	11/11/2023	133	86
	Total	1840	772
	Mean	115	48

### Participants

Interested RTs could learn of the ASPRONET seminars via the IAEA website, or through an email notification, containing time, date and theme details for each seminar, sent approximately three weeks before the seminar date, by the RT leadership team. The email provided a link to a registration form, which generated an invitation to the Microsoft Teams® meeting, which was the videoconferencing platform for all seminars. The registration form allowed basic demographic data to be collected, permitted the maintenance of a cumulative registrants’ email contact list, and was used to send invitations to prospective attendees before each seminar. The RT leadership team invited contributions from attendees, vetted them for anonymization and relevance, supplemented content if required, and compiled a slideshow for each seminar.

### Surveys

**Fig. 1:**
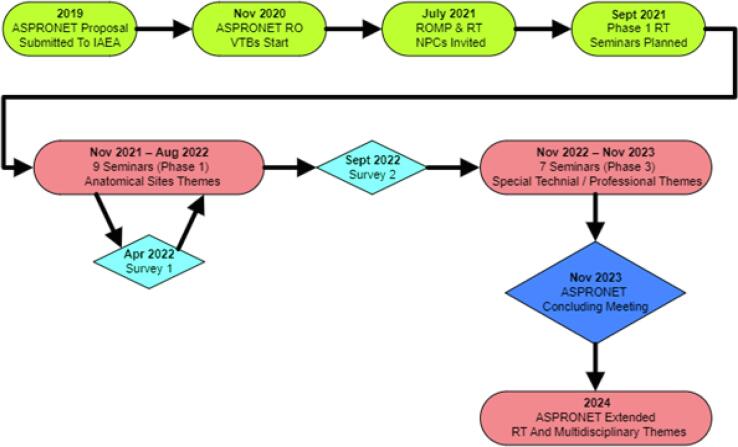
ASPRONET RT Project Development and Administration Timeline.

The email contact list was employed to issue a survey in April 2022 to 88 recipients, evaluating experience with the seminars and attendance barriers. A second registrant survey took place in September 2022 issued to 186 recipients on the email contact list, investigating preferences for format and themes for future seminars, and clinical context details. This survey additionally invited recipients to forward the link to other RTs in their local networks that may not have yet registered or participated in a session. Both surveys were approved by the University of Otago Human Ethics Committee. The second survey’s results prompted design changes for the seven seminars in second phase. Many procedural elements from the first phase were retained, however the focus changed to complex technical practice themes, professional development and RT role evolution. These were held on Saturdays at 05:00 to 06:00 UTC on alternate months. To support attendance, ROs were requested to forward the email notifications of the RT seminars to their professional contacts. Additionally, from seminar 12 onwards, the chairs sent a flyer by email to all contacts one week prior, promoting the upcoming theme, time and date details, and an online registration link. The second phase saw the chairs invite expert presenters in most instances. Fig. 1 displays a timeline of key development and administration points for the entire RAS 6096 RT project.

## Results

### Registrations and attendances

**Fig. 2:**
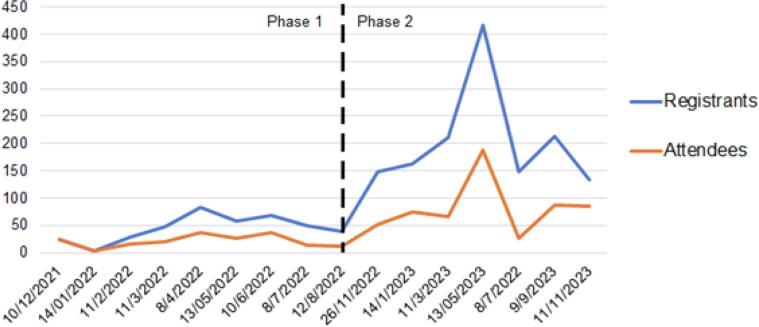
Registrants and Attendees per Seminar.

**Fig. 3:**
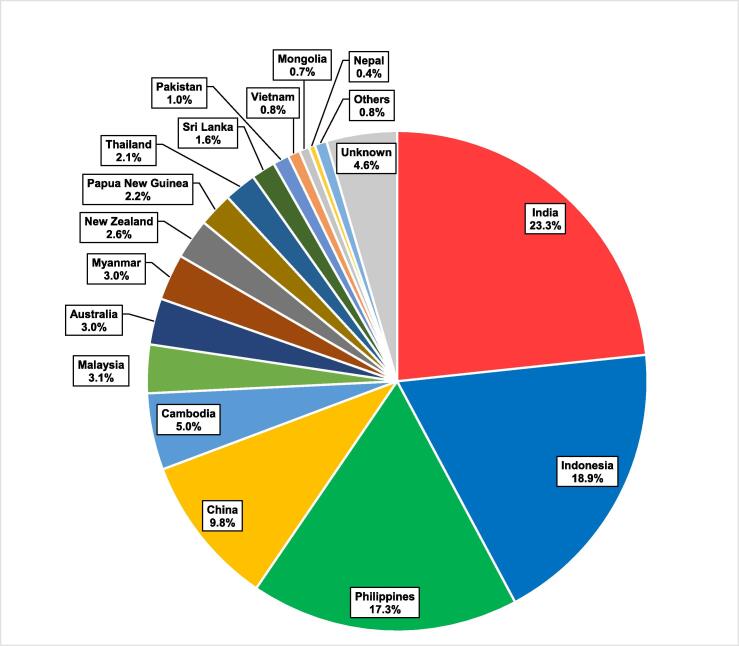
Seminar Attendees by Country.

Table 1 and Fig. 2 show registration and attendance data for the entire series, with a total of 1840 registrants and 772 attendees. A notable increase was observed in the second phase, peaking at 189 attendees in seminar 13. Table 2 shows the professions of registrants and attendees, where RTs comprised 71 % of attendees for the series. Registrants came from 25 countries, while attendees were from 20 countries, 15 of which were Asia-Pacific region LMICs. The registrants email contact list grew to over 500 individuals by the conclusion of the series. Fig. 3 shows the distribution of all attendees by country. The most common nationalities of attendees for the series were India (23.3 %), Indonesia (18.9 %) and the Philippines (17.3 %). Attendance patterns from the suite of countries throughout the series varied, though a decline in attendees from India occurred, while increases from Cambodia, Indonesia, Mongolia, Myanmar, Nepal and the Philippines were observed.

**Table 2:** Clinician Roles of Registrants And Attendees.

**Clinician Role**	**Registrations**	**Attendees**
RT	1262 (69 %)	544 (71 %)
RO	236 (13 %)	88 (11 %)
ROMP	84 (5 %)	28 (4 %)
Other	173 (9 %)	47 (6 %)
Unknown	85 (5 %)	61 (8 %)
Total	1840 (100 %)	772 (100 %)

### Surveys

The first survey yielded 21 responses (23.9 %) from seven countries. It indicated 48 % of attendees had attended two of more of the five seminars run to that point, affirmed seminar durations, frequency, content relevance and audiovisual functionality. 60 % of attendees joined using a smartphone or tablet, and some respondents indicated Zoom® over Teams® as their preferred videoconferencing platform. 67 % reported barriers to attendance due to clinical or personal commitments taking priority at the scheduled time of the seminars. The second survey generated 171 responses (91.9 %) from 10 countries. Interest in continuing the series was expressed by 94 % of respondents. Strong interest was reported for discussion about clinical practice (83 %), learning about techniques and technologies which may be unavailable locally (97 %), and gaining support for clinical practice changes at attendees’ institutions (87 %). 72 % of respondents indicated a desire to continue with monthly seminars, and under 50 % wished to continue the meetings on weekdays, instead preferring Saturdays (73 %) or Sundays (54 %).

## Discussion

### Learning needs and formats

The education, training and professional recognition of radiation therapists is critical to safe and effective radiotherapy. The 21st century has witnessed a rapid uptake of major radiation therapy equipment in LMICs, namely medical linear accelerators and associated software systems, despite resource limitations.[14] These systems enable higher quality treatments and better patient outcomes, but they introduce a substantial increase in clinical practice complexity, with higher technical knowledge and praxis challenges for radiation oncology clinicians including RTs.[15] This, combined with the frequent absence of RT-specific degree programs at universities in LMICs, and professional recognition challenges, create significant clinical education challenges for the limited available personnel. Various substitutes for this shortfall are often limited to vendor training on equipment at the time of installation or upgrades. Vendor training is necessary and useful, but is limited to equipment functionality training and does not usually provide more contextualised learning of integrated clinical practices.[16] A range of initiatives, such as conference attendance and professional organization-funded scientific study visits at well-resourced centres have provided complementary assistance to professionals from LMICs.[17] Voluntary clinical mentoring placements, conducted by High Income Country (HIC) RTs at LMIC centres have helped to advance clinical practice, as well as educating the mentors themselves regarding practice in resource-constrained conditions.[18] Some practical strategies have been identified for collaboration between Asia-Pacific region LMICs and HICs to improve cancer outcomes. These include “Increasing web-based endeavours through virtual tumour boards, web-based advocacy platforms and web-based teaching programs.”[19].

### Project successes

The ASPRONET RT seminar series is an example of a web-based teaching project, which successfully fostered collaborative learning and participant interest across a range of distinct learning needs. Content overtly examined the intricacies of RT clinical practice, facilitated the sharing of experiences, key technical concept application, and reinforced the conceptual connection of interprofessional learning and practice.[20] Results from the first survey and rising attendance numbers through the series suggest that the seminars were educationally valuable and relevant to RTs, as well as ROs. Maximising attendee engagement was a paramount consideration for the chairs throughout the series. The participation of an RT from an LMIC in the coordination team assisted in this, by providing a first-hand perspective and administrative input, which translated to a larger amount of content generation and presentation from LMICs, particularly in the second phase. Promotion of the seminars by ROs, through active endorsement and encouragement of their RT colleagues to attend seminars, and the distribution of reminder flyers by email a week prior, enhanced attendance and interest.

The chairs devised a slide template for the PowerPoint® presentations for all seminars at the start of the first phase, based around a sequence of salient topics that could be applied to each anatomical site. It contained guidelines about the amount and types of content that could be submitted, and allowed monitoring of patient information confidentiality. It also made content compilation easier for the chairs, helped them to plan the pace of each seminar, allowing for sufficient detail in presented materials, and time for questions and discussion. The slide template was simplified during the first phase, and was discontinued in the second phase, where the diversity of complex technical practices themes required unique topic divisions, chosen by the contributors or presenters. The second phase saw the chairs invite expert presenters to speak for entire seminars in most instances. Paediatric and brachytherapy themed seminars were delivered entirely by RTs from LMICs. Some attendees faced information technology barriers during the seminars, including poor Wi-Fi or network connections, difficulty viewing detailed visual content on smartphones, and unfamiliarity with the functionality of Teams®. This improved with repeated attendance, and presenters were encouraged to make their PowerPoint® slide content as accessible as possible, though the chairs could offer no solution to connectivity problems.

### Timeslot challenges

The most significant increase of registrants and attendees occurred when seminars were changed from Fridays in the first phase, to Saturdays in the second phase. This was a direct response to the attendees’ preferences indicated in the second survey. Presumably, it helped attendees to avoid clashes with clinical and personal commitments they may have encountered on weekdays. Increased attendance on Saturdays arguably corresponded with increased attendee engagement. The results of the second survey also indicated a preference to continue with monthly meetings for the series’ second phase. However, this suggestion was not adopted because the more complex themes in the second phase required greater preparation time for both chairs and guest presenters. The chairs also chose not to make recordings of the seminars for offline viewing, to prioritise and promote live engagement, and to avoid the complexities of determining a secure and accessible online hosting location. The first survey confirmed running the seminars at 05:00 to 06:00 UTC on Fridays as suitable, and this was maintained in the second phase on Saturdays. The participating Asia-Pacific region countries in the ASPRONET project extended from New Zealand in the East to Pakistan in the West, with a time zone range of up to eight hours. The chosen timeslot was during waking hours for all attendees, though the convenience in different locations may have varied much more than occurred with the two hour time zone range for the AFRONET project.[13].

### Language challenges

Ideally, in-country training for radiotherapy clinicians in LMICs should be provided in their native language.[16] When applied to remotely delivered online contexts, this challenge is much larger because of the onerous expense and effort it would require to construct and deliver such training resources in numerous distinct languages. IAEA online resources are confined to six languages, and all ASPRONET seminars were conducted in English. The chairs observed some attendees appeared to experience comprehension difficulties with the pace of narration and English vocabulary usage. The leadership team observed that RT attendees from Asia-Pacific region LMICs often possessed lower English competency than their ROMP and RO colleagues. This has been previously noted by others specifically in the Asia-Pacific region context [21] and introduces an additional barrier which may have limited participation in the seminar series.

## Future directions

Improving the education, training, and professional recognition of RTs requires both global and local initiatives. The IAEA continues to work with member states in this domain, through the development of international standards and guidelines, supporting capacity building education and training programs, standardising certification processes for radiation therapy professionals, and providing technical assistance to enhance radiation therapy service delivery.

A multidisciplinary review meeting for the RAS 6096 project was held in November 2023. Many discussion points and other indicators suggested that the series was successful in achieving its primary aims. The meeting endorsed extension of the project with IAEA support through 2024, including the continuation of the RTT seminars. There was consensus that the program will continue beyond 2024. Planning for the third phase of the series has started, with a focus on increasing collaboration, and leadership from LMIC RTs by expanding the coordination team. Inclusion of representatives from Indonesia, the Philippines, Thailand, Pakistan, and Sri Lanka will increase the diversity of experience with different levels of technology. Involvement from nations such as Japan, the Republic of Korea and Singapore, will enhance knowledge transfer, improve seminar quality, broaden the multidirectional understanding of all participating nations’ clinical contexts, and support the sustainability of the ASPRONET RT project.

There should also be a more extensive evaluation of the seminars, including surveying attendee confidence with clinical practice change possibilities, and qualitative feedback about seminars. The 2024 series will place a greater emphasis on promoting interprofessional education through coordination and engagement with a parallel series of multidisciplinary seminars. The activities of dedicated individuals in conducting the ASPRONET project have demonstrated both challenges and opportunities in using online platforms to facilitate information-sharing between RTs in varying clinical circumstances, across a diverse geographic region. Continuation of the project, and its evaluation, will seek to further explore how LMICs can be best supported through online collaborative educational activities. Elements of the project’s structure could produce successful outcomes in comparable online seminar series.

## Declaration of competing interest

The authors declare that they have no known competing financial interests or personal relationships that could have appeared to influence the work reported in this paper.
